# Preliminary experience of an international orthopaedic registry: the ESSKA Paediatric Anterior Cruciate Ligament Initiative (PAMI) registry

**DOI:** 10.1186/s40634-021-00366-7

**Published:** 2021-06-25

**Authors:** Caroline Mouton, Håvard Moksnes, Rob Janssen, Christian Fink, Stefano Zaffagnini, Juan Carlos Monllau, Guri Ekås, Lars Engebretsen, Romain Seil

**Affiliations:** 1 Department of Orthopaedic Surgery, Centre Hospitalier de Luxembourg – Clinique d’Eich, 78 Rue d’Eich, L-1460 Luxembourg, Luxembourg; 2 Luxembourg Institute of Research in Orthopaedics, Sports Medicine and Science (LIROMS), Luxembourg, Luxembourg; 3 Oslo Sports Trauma Research Centre (OSTRC), Norwegian School of Sports Sciences, Oslo, Norway; 4 Department of Orthopaedic Surgery & Trauma, Máxima Medical Center, Eindhoven-Veldhoven, The Netherlands; 5 Orthopaedic Biomechanics, Department of Biomedical Engineering, Eindhoven University of Technology, Eindhoven, The Netherlands; 6 Value-Based Health Care, Department of Paramedical Sciences, Fontys University of Applied Sciences, Eindhoven, The Netherlands; 7 Gelenkpunkt - Sports and Joint Surgery, Innsbruck, Austria; 8 Research Unit for Orthopaedic Sports Medicine and Injury Prevention (OSMI), UMIT Tirol, Hall, Austria; 9 IIa Clinica Ortopedica E Traumatologica, IRCCS Istituto Ortopedico Rizzoli, Bologna, Italy; 10 Hospital del Mar / Hospital Universitari Dexeus, Universitat Autònoma de Barcelona (UAB), Barcelona, Spain; 11 Department of Orthopedic Surgery, Akershus University Hospital, Lørenskog, Norway; 12 Institute of Clinical Medicine, University of Oslo, Oslo, Norway; 13 Department of Orthopaedic Surgery, Oslo University Hospital, Oslo, Norway; 14 Human Motion, Orthopaedics, Sports Medicine and Digital Methods, Luxembourg Institute of Health, Luxembourg, Luxembourg

**Keywords:** Anterior cruciate ligament, Knee, Paediatric, Child

## Abstract

Paediatric and adolescent ACL injuries are an emerging health burden, increasing at a higher rate than in adults. They compromise quality of life, affect knee structure and function, lead to the early development of osteoarthritis and are a serious economic burden due to shortened professional careers and subsequent surgeries. Up to 35% of children and adolescents will experience a second ACL injury and this population particularly at high risk of secondary intraarticular soft tissue degeneration and growth abnormalities. However, there is still a lack of high-quality outcome studies on this specific population and many knowledge gaps persist in the current treatment guidelines. It is currently unknown whether ACL reconstruction in this young population decreases the risk of irreversible secondary intraarticular soft tissue degeneration. Furthermore, it is not known whether return to high or elite level sports after paediatric ACL injury or reconstruction should be recommended. The relatively low number of paediatric ACL injuries seen in each hospital makes it necessary to conduct international multi-centre studies to collect robust data to provide evidence-based guidelines for the treatment of these injuries. The Paediatric Anterior Cruciate Ligament Initiative (PAMI) was thus started by the European Society of Sports Traumatology, Knee Surgery & Arthroscopy and opened for patient inclusion in 2018. This comprehensive overview of the first 2 years of the PAMI registry shows that the project is now well consolidated and accepted by the European orthopaedic community. Future challenges include ensuring additional external funding to ascertain long term sustainability and continuous dissemination of the knowledge acquired in scientific journals.

## Introduction

An anterior cruciate ligament (ACL) injury in paediatric and adolescent populations is a life-long burden both for the paediatric and adolescent athletes and the society. It compromises quality of life, affects future knee structure and function, leads to the early development of osteoarthritis [27] and is a serious economic burden due to potentially shortened professional careers and later surgeries. Paediatric and adolescent ACL injuries are increasing rapidly [7, 20, 25, 29]. This population age group is associated to the fastest growing incidence rate of anterior cruciate ligament injuries [20, 26]. This rise is significantly higher than in adults and places these injuries as an emerging health burden [20].

Many knowledge gaps persist in the current treatment guidelines of paediatric and adolescent ACL injuries [6, 15], leaving the treating physicians with a therapeutic dilemma. Nonoperative treatment has shown to be successful in some patients. However, an association between the delay of surgery and the occurrence of secondary meniscus and cartilage lesions suggests that it may increase the risk of irreversible secondary intraarticular soft tissue degeneration [8]. The limitations in the literature preclude firm conclusions [9, 10]. Paediatric ACL reconstruction surgery is challenging for the orthopaedic surgeon and highly specialised. A variety of surgical techniques have been described in the orthopaedic literature, making it difficult to establish generalizable treatment standards. Given the complex anatomy of paediatric knees, each technique has its individual risks and complications. All of them bear a serious potential to generate secondary growth abnormalities [12]. There is a lack of high-quality outcome studies after surgical treatment and it is furthermore currently unknown whether ACL reconstruction in this young population decreases the risk of irreversible secondary intraarticular soft tissue degeneration. Up to 35% of children and adolescents experience a second ACL injury, be it a retear of their ACL reconstruction or an injury of the unaffected contralateral knee [3, 18]. It remains unknown whether return to high or elite level sports after paediatric ACL injury or reconstruction should be recommended and on which criteria it should be based [4].

The relatively low number of paediatric ACL injuries seen in each hospital makes it necessary to conduct international multi-centre studies to collect sufficient and robust data in order to provide evidence-based guidelines for the treatment of paediatric ACL injuries. The 2018 International Olympic Committee (IOC) consensus statement on prevention, diagnosis and management of paediatric ACL injuries [1] highlighted this multicentre approach as one of the highest research priorities in the field. The Paediatric Anterior Cruciate Ligament Initiative (PAMI) was started by the European Society of Sports Traumatology, Knee Surgery & Arthroscopy (ESSKA) in 2013 with the long-term vision to create a multicentre international registry. The PAMI registry was opened for patient inclusion in late 2018. It represents one of the rare international collaborative studies in orthopaedic surgery and orthopaedic sports medicine. The aim of the present report is to give a comprehensive overview of the PAMI registry, to present preliminary epidemiological data and discuss its future.

## Aims of the initiative

The main purpose of the PAMI registry is to collect data from orthopaedic surgeons who are treating children and adolescents with ACL injuries in order to improve existing treatment algorithms, standardize treatment protocols as well as surgical procedures of paediatric and adolescent ACL injuries on an international level.

The PAMI registry aims to provide important insights into several aspects of paediatric ACL injuries. Initially it will help to assess the magnitude of ACL injuries arising in the paediatric and adolescent population as well as describing current treatment options following a paediatric ACL injury. Understanding of the injury mechanism as well as collective and individual ACL injury risk factors in this specific population may also help establishing a knowledge base for future ACL injury prevention initiatives. The analyses of short-, medium- and long-term clinical outcome will help to improve the understanding of the anatomy, the biomechanics and the development of children’s knees. It will also extend the evidence base on optimal treatment choices by providing important insights into the natural history of ACL injured knees in children, and will allow discriminating those patients needing operative treatment from those who benefit most from a nonoperative treatment. Such knowledge will help to improve rehabilitation strategies in order to reduce the pre-existing high injury recurrence and improve secondary prevention after ACL injury and surgery in this young and active population. The project will provide indispensable data required to identify children at risk for ACL injuries, and to diagnose and treat ACL injuries in children and adolescents based on scientific evidence. Finally, the project will help establishing international treatment guidelines.

## History

The PAMI registry represents one of the rare international collaborative studies in orthopaedic surgery and orthopaedic sports medicine. The initial idea to create an international registry for the treatment of paediatric anterior cruciate ligament (ACL) injuries was born in 2013. The initiative was built on a closed e-survey submitted to ESSKA member and affiliates in July 2013 [17, 21]. The aim of the survey was to describe the treatment options in paediatric ACL injuries. Beyond the fact that it showed the feasibility of the initiative in terms of recruitment capacity, it helped to document that the incidences of paediatric ACL injuries and idiopathic growth disturbances may be higher than previously estimated. Additionally, that there were substantial differences with regard to preferred treatment algorithms, surgical techniques and long-term follow-up procedures. Planning and structuring lasted until 2016 when the development of the PAMI registry was started. In 2018, the PAMI collaboration was officially announced [19] and opened for participation requests during the ESSKA Biennial Congress in May 2018. Inclusion of patients was initiated in December 2018. In February 2021, the number of patients included in the PAMI exceeded 100.

## Stakeholders and overall project organization

The PAMI was initiated, promoted and financially supported by ESSKA and the ESSKA Foundation. Essential financial support as a starting grant was provided by Smith & Nephew and the Olympic Solidarity (OS). The project was undertaken in collaboration with the Sports Medicine Research Laboratory of the Luxembourg Institute of Health (LIH) for its scientific and technical experience. A dedicated steering committee (PAMI steering committee) guarantees that the general objectives of the PAMI project are met, advises ESSKA on strategic decisions regarding the quality assurance and the development of the PAMI registry and is responsible for project related communication and dissemination of results. The daily administrative management is taken care of by a member of the PAMI steering committee acting as the project manager. The project manager’s tasks include, but are not limited to ensure communication with all project stakeholders, assist partner institutions in applying to the PAMI project and ensure that all legal requirements are met. As the PAMI initiative aims at including data from multiple centres, it is open to any institution willing to engage in this project and to provide data on current surgical and non-surgical treatments and their clinical outcomes using a dedicated web portal.

## Patient selection and outcomes

Currently, every patient with a physical activity-related traumatic ACL injury may be recruited for the PAMI provided that the diagnosis is confirmed according to criteria established in the IOC consensus [1] and that the skeletal age at the time of inclusion or at surgery is between 8 and 14 years for girls or 8 and 16 years for boys (as determined on radiographs of the left hand according to the Greulich & Pyle atlas criteria [14]). Patients with knee dislocations, combined ACL-PCL injuries and / or tibial spine fractures are currently excluded. The inclusion of younger patients or patients with bony avulsion or ACL agenesis is currently under discussion.

A data collection platform has been created, the PAMI database or PAMI platform, to carry out a systematic and standardized collection of outcomes judged as relevant to the problem of paediatric ACL injuries. Information collected within the PAMI project currently consist of 5 data categories: patient and injury-related information, clinical examination, treatment and follow-up. Patient data include birth date, sex, height, weight, skeletal age. Other mandatory data are related to injury (date of injury, injured side, previous knee injuries, activity at time of injury, injury mechanism). All centres can also document the results of the Lachman test [24], the pivot shift test [13] and the recurvatum for each patient and injury. Patients are then followed regardless if they are treated surgically or not. In case of a surgical treatment, a full report is requested containing data on the ACL reconstruction (surgical technique, graft, fixations, tunnel …) as well as ACL and associated lesions (other knee ligament injury, menisci and cartilage) and procedures (e.g. extra-articular tenodesis…). The skeletal age at the time of the surgery as well as the clinical examination under anaesthesia (Lachman test, the pivot shift test and recurvatum) can also be specified. Finally, patients are contacted every year to fill in the Paediatric International Knee Documentation Committee Subjective Knee Form in Children (Pedi-IKDC) [16] and the Pediatric Activity Rating Scale (HSS Pedi-FABS) [11]. These questionnaires have been shown to be adequate for children with knee ligament injuries [5]. They are currently available in English, German, French, Norwegian, Italian and Dutch. The foreseen follow-up is of 30 years for each patient. Any new ACL injury (i.e. to the same or opposite knee) that may occur during the follow up of the patient can be documented so as to know the recurrence rate of ACL injuries in this population.

The PAMI steering committee and partner institutions are currently discussing to develop the long-term outcomes such as imaging and its variables of interest (i.e. growth disturbance, tibial slope…). Although growth disturbances are rare, they are a serious risk [12] and require a regular patient monitoring in order to detect any leg length discrepancy or malalignment which may be the result of a growth arrest, an over- or undergrowth process. Other data on knee function such as the results of functional performance tests, of isokinetic force tests of knee extensor and flexor muscles, of knee joint laxity measurements may also be implemented.

## PAMI database

All data are entered in a secured website with a strictly limited access to PAMI participating centres. The compliance of the project and platform with the General Data Protection Regulation (GDPR) 2016/679 has been previously explained in a specific book chapter [22]. In summary, a secured communication protocol has been implemented to secure data communication to and from the PAMI database. The system works with a two-factor authentication solution: the site coordinator enters his username/password and receives a numeric code via SMS (Short Message Service) on his or her personal cell phone to be able to enter the platform.

All included data are pseudonymized to ensure maximal data protection and avoid legal issues related to data transfer between different European countries. Patient identification information is replaced by a patient ID and data cannot be attributed to a patient. As a consequence, each site-coordinator manages a correspondence table between patient ID information and a primary key generated by the system to ensure long-term follow-up of each patient.

## Preliminary epidemiological data

**Fig. 1:**
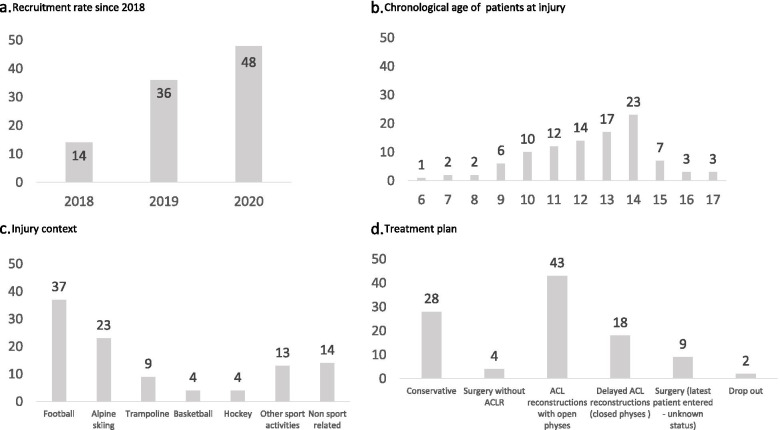
Preliminary epidemiological data from PAMI. **a** Number of patients recruited in the PAMI since 2018 (2 patients recruited in 2021). **b** Chronological age of patient at injury (*n* = 100 patients). **c** Injury context (*n* = 104 injuries). **d** Treatment (*n* = 104 injuries)

From the first inclusion in October 2018 to February 2021 (Fig. 1a), 100 patients (71 males / 29 females) from 7 institutions (Austria, Italy, Luxembourg, Netherlands, Norway, Spain) were included in the PAMI registry. These patients sustained 104 ACL injuries (4 patients with 2 injuries: 3 contralateral ACL injuries and 1 graft rupture). The median chronological age at injury was 13 (interquartile range: 3) years (Fig. 1b). Most injuries occurred during physical activities (87%) mainly while playing football (*n* = 37), during alpine skiing (*n* = 23) or trampoline (*n* = 9) (Fig. 1c).

At the time of the analysis, there were 28 ongoing nonoperative treatments, 4 patients with knee surgery without ACL reconstruction, 70 ACL reconstructions and 2 drop out (patients moved into another hospital; Fig. 1d). Time from injury to surgery reached a median of 6 months. Patients undergoing surgery were further separated into 2 groups: ACL reconstructions with open physes (*n* = 43; mean age = 13 ± 2) and ACL reconstructions delayed until the end of the growth period (closed physes – *n* = 18; mean age = 15 ± 1). Overall, 36 patients out of 61 (59%) presented with a meniscal tear (5 bimeniscal tears). In ACL reconstructions with open physes, semitendinosus and/or gracilis graft were preferred (72%) over quadriceps tendon (28%). Extraarticular ACL tenodesis were performed in 40% of patients. Lesions to the medial meniscus were observed in 7 knees (16%), of which 6 were repaired. Lateral meniscus lesions were observed in 19 knees (44%), of which 9 were repaired. In delayed ACL reconstructions, quadriceps tendon (44%), semitendinosus and/or gracilis (39%) and patellar tendon (17%) were used. Extraarticular ACL tenodesis were performed in 33% of patients. Lesions to the medial meniscus were observed in 8 knees (44%), of which 6 were repaired. Lateral meniscus lesions were observed in 6 knees (33%), of which 3 were repaired.

## Preliminary experience and future perspectives

This is the first time that a comprehensive overview of the PAMI registry is provided, together with preliminary epidemiological data. The PAMI registry is one of the rare international collaborative studies in orthopaedic surgery and orthopaedic sports medicine. A similar project in children named “Pediatric ACL: Understanding Treatment Options (PLUTO)” has been started in 2016 and is conducted in 10 centres in North America with close to 800 patients (August 3, 2020) enrolled so far.

While being in line with the research priorities highlighted by the 2018 IOC consensus statement on prevention, diagnosis and management of paediatric ACL injuries [1], these initiatives represent an organizational challenge and many barriers may interfere with their set up. First of all, the overall project organisation and study protocol are to be consolidated. While the idea of the PAMI was born in 2013, first patient inclusion could only be achieved in 2018. Many prerequisites were indeed required in order to launch such an international multicentre initiative. These are similar to the planning of a patient/disease registry. According to the published user guides to evaluate patient outcomes [23], it was important to articulate the purpose, identify key stakeholders and assess the feasibility and sustainability of the future registry. Then, a registry team, previously mentioned as the steering committee, was built in order to establish the governance and oversight plan, define the scope and rigor needed, define the targeted population, dataset and outcomes, develop the study protocol and assess the follow-up of the project plan. Likewise, innovative technological solutions had to be implemented. In case of the PAMI, project development included the creation of a specific IT platform which took an additional year. Setting up multicentre initiatives thus represents years of preparation and the related organisational process is rarely linear. Many of the previously cited steps may occur in parallel and should be periodically (re)evaluated to ensure that the objectives are being met. For example, in the case of ACL injuries in paediatric and adolescent populations, treatment practices may rapidly evolve over years. This requires that the content of the PAMI database is regularly adapted (i.e. adding additional field of data if a new surgical technique is used or a new possible answer in a drop-down list) in order to capture the latest trends.

Major difficulties encountered during the first years of the PAMI included funding, ethical approval and recruitment. Unstable funding remains the main threat in every research project one may encounter [28]. Currently, long term sustainability of the PAMI must be ascertained by additional external funding. The interest of partner institutions is no longer to be proven, but project continuity will have to be constantly maintained through regular feedback to stakeholders, common publications and networking initiatives. The first years of the project were decisive to consolidate the project in its long term although patient recruitment started slowly. The main reasons behind this were that ethical and legal requirements had to be fulfilled before any patients could be entered in the database. Despite the standardisation of European legislation on clinical trials and data protection, the ethical aspects probably remain a major weakness of any international multi-centre studies. The administrative burden for each partner institution was high as they had to seek ethics clearance to their local or national ethics committee in accordance with their national laws and regulations. This process is highly variable in the various countries of the EU and was also slowed down due to required translations of patient information, consent and questionnaires (Pedi-IKDC and HSS Pedi-FABS) which were translated through a standardised process [2]. Beginning of 2021, 8 centres were actively recruiting patients with a continuous increase of patient inclusion observed over time. Although the current results are limited to 100 patients, we are confident that the recruitment will continue to grow exponentially with the increase of partner institutions. Seven additional centres from Italy, Greece, France and UK are indeed in the process of ethics clearance in order to join the initiative.

The feedback of recruiting centres had a significant impact on the evolution and consolidation of the PAMI registry. It has helped to adapt the PAMI database to enhance user’s experience with the PAMI web portal as well as to consolidate data accuracy and completeness, two of the major data quality dimensions according to methodological guidelines for registries [28]. This was only possible with an extensive and continuous communication between the different partners. Communication between the steering committee and partner or interesting institutions has now been planned and standardised and includes (1) a yearly newsletter to increase awareness about the initiative and recruit new centres, (2) guidelines provided to partner institutions to ensure data quality and proper use of the PAMI platform, (3) 2 participants newsletter per year including latest relevant publications and the evolution of the recruitment and database to keep all partners motivated, (4) a common workshop every year to discuss about the difficulties around the project and its perspectives. As an example, during the last workshop in October 2020, inclusion/exclusion criteria were discussed again as well as the possibility to extend the content of data gathered within the project (i.e. assessment of long-term outcomes). In addition to this communication strategy, the PAMI managers will organise in 2021 the first monitoring of PAMI data and start to provide yearly personalised feedbacks to partner institutions on their data in order to ensure the quality of data. The PAMI steering committee will make it a priority for the next years to continue to improve the PAMI registry. This will be achieved through the organisation of periodic critical evaluations to ensure that the PAMI objectives are met and up to date or to be able to quickly react and adapt the initiative in line with the relevant published scientific literature within the field.

## Conclusion

This comprehensive overview of the first 2 years of the PAMI experience shows that the project is now well consolidated and accepted by the orthopaedic community. The strategy to ensure data quality, communication with partner institutions and dissemination of results is now well established. Future challenges include ensuring additional external funding to ascertain long term sustainability of the PAMI and continuous dissemination of the knowledge acquired in scientific journals. These continuous efforts are needed to improve existing treatment algorithms, standardize treatment protocols and improve rehabilitation strategies to reduce the pre-existing high injury recurrence in paediatric and adolescent ACL injuries and surgery on an international level.

## Data Availability

Not applicable.

## Abbreviations

ACL: Anterior cruciate ligament
ESSKA: European Society of Sports Traumatology, Knee Surgery & Arthroscopy
GDPR: General Data Protection Regulation
HSS Pedi-FABS: Pediatric Activity Rating Scale
IOC: International Olympic Committee
LIH: Luxembourg Institute of Health
MRI: Magnetic resonance imaging
OS: Olympic Solidarity
PAMI: Paediatric Anterior Cruciate Ligament Initiative
Pedi-IKDC: Paediatric International Knee Documentation Committee
PCL: Posterior cruciate ligament
PLUTO: Pediatric ACL: Understanding Treatment Options
